# ACTIVITY EFFORT, SELF-MANAGEMENT AND INFLAMMATION IN OLDER MEXICAN AMERICANS WITH OSTEOARTHRITIS

**DOI:** 10.1093/geroni/igz038.612

**Published:** 2019-11-08

**Authors:** Tracie C Harrison, Lauren Thill, Bianca R Schmidt, Shelley Blozis

**Affiliations:** 1 The University of Texas at Austin, Austin, Texas, United States; 2 The University of TX, Austin, Texas, United States; 3 The University of Texas, Austin, Texas, United States; 4 University of California, Davis, California, United States

## Abstract

We extend our ongoing investigations of the health effects of activity effort among Mexican Americans (MA) with mobility limitations, specifically those with osteoarthritis (OA) (Harrison, 2009). Our previous research linked activity effort with co-morbidity and social participation in women with mobility limitations, finding significant variations between Non-Hispanic White and MA with physical disabilities. This bio-behavioral study takes the next step by examining the relationships between inflammatory measures (TNF-alpha & CRP), Mexican American-specific self-management behaviors (MA-SM), and activity effort (AE) in a sample of MA men and women. Over 5 months, 62 men and women, age 40 to 83, provided survey responses, blood, and saliva for analysis. After ensuring reliability of measures, we used Pearson correlations to provide initial associations. Findings indicated a significant negative correlation between AE and TNF-alpha (-.376, 0.005), which linked behaviors to inflammatory response; and between MA-SM and AE (-.254, 0.05), which linked the self-management to the behavior. These findings provide support for the biological impact of perceived activity effort on inflammation, as well as the positive effects that Mexican American specific self-management activities might have on health.

